# Students benefit from developing their own emergency medicine OSCE stations: a comparative study using the matched-pair method

**DOI:** 10.1186/1472-6920-13-138

**Published:** 2013-10-07

**Authors:** Wolfgang Heinke, Daisy Rotzoll, Gunther Hempel, Michaela Zupanic, Patrick Stumpp, Udo X Kaisers, Martin R Fischer

**Affiliations:** 1 Department of Anaesthesiology and Intensive Care Medicine, University of Leipzig, Liebigstrasse 20, Leipzig 04103, Germany; 2 Training Clinic of the Faculty of Medicine, University of Leipzig, Liebigstrasse 27, Leipzig 04103, Germany; 3 Faculty of Health, University of Witten/Herdecke, Alfred-Herrhausen-Straße 50, Witten 58448, Germany; 4 Department of Diagnostic and Interventional Radiology, University of Leipzig, Liebigstrasse 20, Leipzig 04103, Germany; 5 Department of Medical Education, Munich University Hospital, Ludwig-Maximilians-University Munich, Ziemssenstraße 1, Munich 80336, Germany

**Keywords:** OSCE, Emergency medicine, Undergraduate education, Assessment of training

## Abstract

**Background:**

Students can improve the learning process by developing their own multiple choice questions. If a similar effect occurred when creating OSCE (objective structured clinical examination) stations by themselves it could be beneficial to involve them in the development of OSCE stations. This study investigates the effect of students developing emergency medicine OSCE stations on their test performance.

**Method:**

In the 2011/12 winter semester, an emergency medicine OSCE was held for the first time at the Faculty of Medicine at the University of Leipzig. When preparing for the OSCE, 13 students (the intervention group) developed and tested emergency medicine examination stations as a learning experience. Their subsequent OSCE performance was compared to that of 13 other students (the control group), who were parallelized in terms of age, gender, semester and level of previous knowledge using the matched-pair method. In addition, both groups were compared to 20 students who tested the OSCE prior to regular emergency medicine training (test OSCE group).

**Results:**

There were no differences between the three groups regarding age (24.3 ± 2.6; 24.2 ± 3.4 and 24 ± 2.3 years) or previous knowledge (29.3 ± 3.4; 29.3 ± 3.2 and 28.9 ± 4.7 points in the multiple choice [MC] exam in emergency medicine). Merely the gender distribution differed (8 female and 5 male students in the intervention and control group vs. 3 males and 17 females in the test OSCE group).

In the exam OSCE, participants in the intervention group scored 233.4 ± 6.3 points (mean ± SD) compared to 223.8 ± 9.2 points (*p* < 0.01) in the control group. Cohen’s effect size was *d* = 1.24. The students of the test OSCE group scored 223.2 ± 13.4 points.

**Conclusions:**

Students who actively develop OSCE stations when preparing for an emergency medicine OSCE achieve better exam results.

## Background

Emergency medicine is an area where successful treatment hinges on clinical competence and practical skills. Since doctors are expected to perform efficiently in emergency medicine, practical essentials must be mastered by every doctor. One way of ensuring the necessary expertise is to test whether medical students meet the required standards of emergency medicine in practical exams like the objective structured clinical examination (OSCE) [1].

OSCEs are used to examine practical clinical skills, the application of procedural knowledge, and under certain circumstances the existence and honing of medical attitudes [2-4]. Moreover, OSCEs are a popular examination format among students [1,5,6]. Despite these advantages, OSCEs are not yet routinely used on medical students (including emergency medicine) in Germany [7]. Instead, students’ knowledge of emergency medicine continues to be solely assessed by means of multiple choice exams at 89% of German medical faculties [8,9].

OSCEs in emergency medicine are rarely used because they are labour-intensive and require large quantities of materials [8]. Numerous organizational and logistical problems need to be solved prior to an OSCE, such as devising a sufficient number of OSCE stations which reflect the syllabus and educational aims. Furthermore, the execution of an OSCE needs to be precisely planned: enough trained examiners must be on hand, the exam stations need to be prepared (e.g. provided with suitable simulation materials and consumables), and the results need to be evaluated in due time.

The time and resources required for a medical school to prepare OSCEs could be reduced if students were involved in their planning. It has been reported that questions in MC exams which were developed by students improved their training and enabled them to achieve higher scores in both oral and written examinations [10-12]. This positive effect is mainly accounted for by students’ knowledge being activated by generating their own questions [10]. If this principle were applied to more complex types of examinations such as OSCEs, perhaps an even greater improvement in students’ learning and assessment performance could be achieved.

Studies are needed to ascertain how students’ scores in a regular OSCE exam are affected if they voluntarily develop OSCE stations. If it turns out that developing OSCE stations results in better exam marks by activating their knowledge and skills, this would be a good argument for systematically involving students in the preparation of OSCEs. It would reduce faculties’ time and resources spent on preparing OSCEs while students could demonstrate improved practical skills in their exams.

The aim of this study was to investigate whether the voluntary development of OSCE stations by students leads to better examination results. Given students’ improved MC exam results after producing their own MC questions, we expected to see a similarly positive impact on an OSCE after the voluntary development of OSCE stations.

## Methods

### Ethics committee, data protection

Prior to the study, ethics approval was applied for from the Faculty of Medicine of the University of Leipzig’s ethics committee, who decided this study did not require special approval. Even so, for reasons of data protection, each participant gave written consent to their participation and to the anonymous evaluation and publication of the findings.

### Emergency medicine on the curriculum in Leipzig

Emergency medicine is taught in Leipzig starting in the seventh semester. The syllabus is based on the learning objectives specified by the German Society of Anaesthesiology and Intensive Care [13]. Training comprises a series of 20 lectures on emergency medicine followed by a four-week PBL course (problem-based learning). This is designed to ensure that students first take theoretical instruction before undergoing extensive practical, clinically oriented training on the PBL course. During the Emergency Medicine PBL course, students work through eight paper and online cases in small groups facilitated by specially trained tutors. In addition, intensive training in emergency medical techniques takes place during the periods of practical training in Human Rescue, Emergency Room Management, Emergency Imaging and Cardiopulmonary Resuscitation under the guidance of university lecturers and emergency services staff. Parallel to this, during the four-week Emergency Medicine PBL course, students have the opportunity to consolidate their practical skills in additional voluntary training sessions run by faculty-trained student tutors.

Until the 2010/11 winter semester, emergency medical training (the series of lectures and the PBL course) concluded with an MC test. In this particular semester, the MC exam was for the first time followed by an emergency medicine OSCE in order to test students’ practical skills.

### Development of an emergency medicine OSCE

In the 2010/11 winter semester, an OSCE in emergency medicine was held for the first time at the University of Leipzig’s Faculty of Medicine. The examination topics were chosen and the OSCE stations devised by a team of anaesthesiologists, emergency physicians, cardiologists, radiologists, paediatricians and students based on both learning objectives in the syllabus [13] and a needs analysis of the targeted learners [14]. The content of the individual stations was worked out by the team’s medical members and verified by representatives of the disciplines involved. Scenarios and checklists for the individual stations were compiled jointly. All stations of the emergency medicine OSCE were tested by a group of 20 students before emergency medicine training started in order to check and, if necessary, modify the stations. The OSCE stations used in the emergency medicine exam are listed in Table 1.

**Table 1:** Brief description of the OSCE stations

**Station**	**Setting,task**
Basic life support	• An unconscious person (mannequin) has been discovered at a building site
	• Basic resuscitation
Advanced life support	• You are a member of a resuscitation team and are required to assist resuscitation (mannequin)
	• Pulse analysis, electrotherapy and drug treatment of cardiac arrest
Insertion of a peripheral venous catheter in a polytrauma patient	• Providing intravenous access in an unconscious polytrauma patient (mannequin arm)
	• Correct insertion using the right size peripheral venous catheter, selection of suitable infusion solutions
Chest pain	• 51-year-old patient with retrosternal pain at A&E
	• Establishing medical history, ECG evaluation, administration of drugs
Bag valve mask ventilation	• You are a member of a resuscitation team and responsible for securing the airway during resuscitation
	• Demonstrating the correct performance of bag valve mask ventilation (airway mannequin)
Polytrauma: helmet removal and neck immobilization	• Motionless motorcyclist (mannequin) with helmet still on and visor closed after colliding with a tree
	• Demonstration of the correct removal of the helmet and neck immobilization using Stifneck®
Newborn resuscitation	• Treatment of a baby born by emergency Caesarean (mannequin) in hospital
	• Evaluation and stimulation (if necessary resuscitation) of the baby
Focused assessment with sonography for trauma	• Polytrauma management in the casualty room (A&E, sonography training system)
	• Performance of emergency sonography showing Morison’s pouch, Koller’s pouch, epigastric region and heart, urinary bladder
Preparing and carrying out blood transfusion	• Postoperative tachycardia and anaemia in a coronary disease patient in the recovery room
	• Preparing and carrying out transfusion, demonstration of ABO compatibility test
ECG	• Patient chest pain on the left (mannequin)
	• Recording an ECG, arrangement of further diagnosis

The 10 stations described in the table were used in the summative OSCE. These stations were developed by the responsible teaching stuff of the faculty. However, some similarities exist compared to the stations developed by the students (see also Table 2).

### Study design

Originally, two groups of students, an intervention group (n = 13) and a control group (n = 13), were formed in a matched-pair design to test the working hypothesis. Additionally, 20 students who had tested the emergency medicine OSCE prior to regular emergency training were included in the study and compared to the intervention group (test OSCE group, n = 20).

### Intervention group

A total of 13 students (8 females, 5 males) were recruited for the intervention group who were in their seventh semester of medicine at the Faculty of Medicine at the University of Leipzig. These students voluntarily developed OSCE stations under the guidance of trained teaching personnel (first author, W.H.). At this time, none of the 13 students had any personal experience of either emergency medicine or the OSCE format.

Two workshops were held to explain the structure of an OSCE, the examiner checklists and scoring. The students were asked to form groups in order to develop OSCE stations together. They set up eight OSCE stations to be trialled at Leipzig SkillsLab (see Table 2). The development process was supported by two authors (W.H., P.S.) of this study. Students received feedback on the stations they had developed: on medical aspects from the relevant teaching staff and on educational aspects from lecturers with a Master’s degree in Medical Education (W.H., P.S.).

**Table 2:** Description of the OSCE stations developed by students

**Station**	**Setting,exercise**
Airway/intubation	• Discovery of a 50-year-old man showing no signs of life
	• Intubation to secure the airway
Advanced life support	• 60-year-old patient at the ward with cardiovascular arrest
	• Operation of a defibrillator
Peripheral venous access	• Insertion of a peripheral venous catheter (mannequin arm) in a 75-year-old female patient at an internal medicine ward
	• Assessment, preparation and insertion of intravenous access
Angina	• A 60-year-old man arrives at A&E by ambulance with severe chest pains
	• Focused case history, differential diagnosis of chest pain, clinical examination for chest pain
Bag valve mask ventilation	• Unconscious female on the floor
	• Demonstrating the correct performance of bag valve mask ventilation (airway mannequin)
Neck immobilization	• 26-year-old woman still conscious after jumping out of a window
	• Correct immobilization of the neck using Stifneck®
Cardiopulmonary resuscitation of a baby	• Anxious mother calls the doctor at the paediatric ward because her four-month-old child is not responding
	• Taking vitals of a baby (mannequin), resuscitation (one- and two-person)
Central venous catheter	• Patient with hypertonia and tachycardia suffering acute pancreatitis at A&E
	• Demonstration of insertion points of the central venous catheter, explanation of the procedure

Eight stations where the students tested each other were set up at Leipzig Training Clinic. The students also marked each other using the examiner checklists compiled before testing the stations that they had devised themselves in a simulated OSCE. Please note, none of these OSCE stations developed by students were used during the summative OSCE. Stations used in the summative OSCE were developed by the responsible teaching staff.

All members of the intervention group used the eight OSCE stations they had developed to test each other’s knowledge of emergency medicine in a simulated OSCE. For this purpose, on the day of the simulated OSCE the students were given additional training in order to take on the role of the expert rater. This OSCE was held under the supervision of the responsible teaching staff (including some of the authors: W.H., G.H., D.R. P.S.) one week before the practical parts of students’ regular emergency training started. The participants of the intervention group had therefore not been trained in practical emergency medicine but had merely had the opportunity to attend emergency medicine lectures beforehand, where both their knowledge and the stations they had created were tested.

### Control group

Thirteen students who took part in the first emergency medicine OSCE in the 2010/11 winter semester made up a control group in order to compare the exam results and test the working hypothesis. Instead of being chosen at random, the members of the control group were selected by using the matched-pair procedure in order to ensure their prior knowledge of general and emergency medicine were comparable to the intervention group. Students were therefore chosen from the same semester who had achieved the same score in the emergency medicine MC exam. Since there were several suitable control group candidates among the 321 OSCE participants, their gender and age were also parallelized (Table 3). This enabled each member of the intervention group to be matched to a student in the control group, producing a series of thirteen matched pairs.

**Table 3:** Comparison of the scores of the intervention group and the control group

**Intervention group**					**Control group**			
**Stud.**	**Gender**	**Age**	**MC**	**OSCE**	**Gender**	**Age**	**MC**	**OSCE**
1	M	22	34	226.5	M	23	34	209.5
2	F	24	34	229.5	F	22	34	221.5
3	M	23	33	235.5	M	23	33	229.5
4	M	23	33	240.0	M	22	33	228.5
5	F	28	31	235.5	F	24	31	224.0
6	M	27	30	232.0	M	26	30	216.5
7	F	22	29	241.0	F	22	29	227.5
8	F	30	27	227.0	F	32	27	221.0
9	M	23	27	221.0	M	23	27	228.5
10	F	24	27	243.0	F	24	27	205.5
11	F	21	26	231.0	F	21	26	239.0
12	F	25	26	236.5	F	24	26	232.5
13	F	24	24	235.5	F	24	24	225.5
**Mean**		**24**.**3**	**29**.**3**	**233**.**4**		**24**.**2**	**29**.**3**	**223**.**8**
(SD)		(2.6)	(3.4)	(6.3)		(3.4)	(3.2)	(9.2)
Mean comparison. *t*-test for paired samples						p > 0.05	p > 0.05	p < 0.001
Effect size (Cohen’s *d*)						d = 1.24		

Demographic data and exam results of the student participants in the intervention group and the control group (Stud. = student number; SD = standard deviation, F = female, M = male). The maximum scores that could be reached were 250 points in the OSCE and 40 points in the MC test.

#### Test OSCE group

This group served as control to exclude the advantage of the intervention group, who became familiarized with the OSCE format by trying out their stations in a simulated OSCE. Therefore, we compared the intervention group to a group of students who completed the emergency OSCE before regular emergency training started (see above). This group was formed for two reasons. Firstly, we wanted to test the suitability of our OSCE. Secondly, we wanted to demonstrate the efficacy of our four weeks’ practical training in emergency medicine. Therefore, 20 voluntary students of the 7th semester took the emergency OSCE one week before regular emergency training started (i.e. at the same time as the student-authored OSCE stations were tested by the intervention group in their simulated OSCE). In order to avoid a familiarity bias with the OSCE format, we compared the OSCE scores of the intervention group to the scores of these 20 students (= test OSCE group) obtained during the regular exam OSCE.

### OSCE

The emergency medicine OSCE consisted of ten stations (Table 1). Eight stations were provided with simulation models (insertion arm for peripheral venous access, a mannequin for basic and advanced life support, a sonography torso, a mannequin of a newborn, an ECG training mannequin, and a mannequin for neck immobilization) while two stations were furnished with examination and diagnostic materials (ECG evaluation, blood transfusion).

The OSCE circuit was set up twice for the exam, enabling 20 students to be examined at once. The study participants were randomly assigned to one of the OSCE circuits by the staff of the registrar’s office, who were not involved in the study (i.e. the circuits were randomly filled with participants of the three study groups; participants completed either circuit 1 or 2). The exam was held at Leipzig SkillsLab since it contains a series of differently equipped rooms (outpatient room, sonography room, etc.), allowing clinical events to be realistically simulated. None of the stations developed by the intervention group were used for this summative OSCE.

At each station, the students had five minutes in which to perform the tasks set. One minute was allowed to move from one station to the next. Students were also given a minute before the first station to familiarize themselves with the tasks. The total duration of the exam was hence 60 minutes. A starting signal and a time’s up signal were sounded at each station. Each day, 120 students were examined by junior doctors, specialists and consultants from various departments of the Faculty of Medicine. Examiners at the individual stations were chosen based on their specializations. Radiologists for instance were in charge of the emergency sonography station (FAST), trauma surgeons manned the neck immobilization station, and paediatricians handled the newborn resuscitation station. They were given thorough training in their examining duties beforehand. This began with a 45-minute theoretical training session on OSCE for all expert raters one week before the OSCE. On the day before the OSCE, once the OSCE course had been completely set up, the raters were trained at their stations. A responsible lecturer demonstrated the performance expected of examinees at each station. The raters were also shown how to use the checklists or to make a global rating. On stations with global rating, the raters were given written guidelines on how to award points in order to minimize interrater differences. The raters were given a final briefing 30 minutes before the start of the OSCE. This training was intended to minimize raters’ impact on the test results. In addition, after each round of tests, the points awarded at each station were reviewed by the first author of this study (W.H.). If any anomalies were found (e.g. above-average or exceptionally low scoring), the raters were given additional training.

Students’ performances were assessed by the examiners with checklists [15,16]. Usually five tasks had to be performed at each station. As the maximum score at each station was 25, the maximum possible total score of the OSCE was 250.

Depending on the station and the exercise, scoring was done by using either a global rating or a checklist rating [16]. The OSCE was carried out as a compensatory examination (i.e. examinees did not have to pass every station – instead, their exam grade depended on their total score) and the pass rate was set at 60% [16].

The complete OSCE was tested before regular emergency training was started by 20 students (test OSCE group, see above) and faculty staff.

### Statistics

The data were logged and processed in Microsoft Excel. Afterwards, group comparisons were carried out. In order to ascertain the effect of students actively developing OSCE stations, the total scores in the OSCEs of the three groups were compared by means of an analysis of variance followed by Bonferroni correction for multiple comparisons. Moreover, a direct comparison between the intervention and control groups was carried out as originally planned with respect to matched pairs design, i.e. the OSCE scores of both groups as well as the results at the individual stations were compared by means of Student’s *t*-test if the data were normally distributed and the Mann–Whitney *U* test for non-normally distributed data. Results below a significance level of *p* < 0.05 were rated as significant. Finally, Cohen’s d as effect size was calculated in order to compare the scores achieved in the OSCE. The data were evaluated using the software suites PASW Statistics 18.0 and Sigmastat (Version 2.0).

## Results

### Demographic data and previous knowledge

The average age of the students in the intervention group was 24.3 ± 2.6 (mean ± standard deviation) years. As a result of the matching procedure, the mean age of the control group (24.2 ± 3.4 years) and its gender distribution were no different from the intervention group (*t*-test for paired samples, *p* > 0.05; Table 3). The students of the test OSCE group had the same mean age (24 ± 2.3 years) but significantly different gender distribution (3 male, 17 female vs. 5 male, 8 female). Comparing their MC results by means of a one-way ANOVA revealed no differences between the three groups (*p* > 0.05). Students in the intervention group scored 29.3 ± 3.4 points, those in the control group achieved 29.3 ± 3.2 points, while the test OSCE group achieved 28.9 ± 4.7 (see Table 4). In addition, a *t*-test for paired samples comparing the intervention group and the control group with respect to the matched pairs design revealed no differences between the two groups (*p* > 0.05; Table 3).

**Table 4:** MC and OSCE scores of the investigated groups

	**Intervention group(n = 13)**			**Control group(n = 13)**			**Test OSCE group (n = 20)**		
	**Mean ± SD**	**Max**	**Min**	**Mean ± SD**	**Max**	**Min**	**Mean ± SD**	**Max**	**Min**
**OSCE**	233.4 ± 6.3	243	221	223.8 ± 9.2	239	205.5	223.2 ± 13.4	239.5	179.5
**MC**	29.3 ± 3.4	34	24	29.3 ± 3.2	34	24	28.9 ± 4.7	36	20

MC and OSCE scores of the Interventions group, Control group and test OSCE group (SD = standard deviation, Min = minimum score, Max = maximum score). The differences between the intervention group and control group as well as between the test OSCE group are significant (p < 0.05).

### Comparison of overall results

During the OSCE, the students in the intervention group scored 233.4 ± 6.3 points, on average 9.6 higher than their counterparts in the control group (223.8 ± 9.2). The students in the test OSCE group scored 223.2 ± 13.4 points in the regular exam OSCE. A one-way ANOVA with factor group revealed significant differences between the three groups (*p* = 0.024). The pairwise multiple comparison procedure (Bonferroni *t*-test) showed significant differences between the intervention group and the test OSCE group (*p* = 0.031) and nearly significant differences between the intervention group and control group (*p* = 0.082).

Again, we compared the scores of the intervention group and the control group by means of the *t*-test for paired samples because these two groups were matched regarding the number of participants, age and gender. Table 3 displays these results. The mean difference of 9.6 points in the OSCE is significant (*t*-test for paired samples, *p* < 0.01) with a large effect size of Cohen’s *d* = 1.24 (d ≥ 0.8 = large effect).

The other participants of the emergency medicine OSCE of the 7th semester (n = 275) who were not in any of the three groups scored on average 216.6 ± 16.5 points.

Since only some of the student-authored stations were similar to stations that were used in the OSCE, the individual stations were compared for the intervention group and the control group. The aim was to determine whether the learning improvements stemming from station development were only related to the specific stations used in the OSCE or whether devising OSCE stations as a whole improves students’ mastery of practical emergency medicine. Note that because the students and teaching staff developed OSCE stations independently, the similarities between the simulated OSCE and the exam OSCE stations were coincidental.

### Scores at the individual OSCE stations

Means (± SD) of the scores achieved at the 10 stations of the OSCE compared between the intervention group (blue) and the control group (orange). Statistical differences are marked by an asterisk. They can be substantiated for the venous access and basic life support stations (*p* < 0.05). The maximum possible score at each station was 25 points.

**Figure 1:**
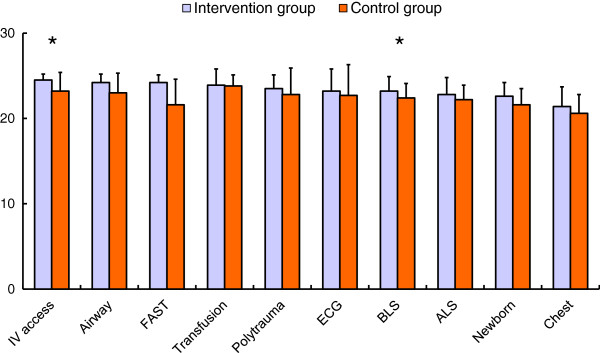
**Scores at the individual OSCE stations.** Significant differences are marked by an asterisk.

Figure 1 shows a clear trend with the students in the intervention group scoring better than the control group at all stations. Although this effect can be statistically demonstrated for the basic life support (*p* = 0.043) and venous access (*p* = 0.037) stations, the differences at the other individual stations were not significant (*t*-test for paired samples at normal distribution, Mann–Whitney *U* test with non-normal distribution, *p* > 0.05).

## Discussion

This paper explores the hypothesis that students perform better in OSCEs if they develop OSCE stations. This hypothesis was confirmed since the students in the intervention group achieved significantly higher exam marks than those in both comparison groups (the control group and the test OSCE group). As the participants in all groups had scored the same results in the previous MC exam in emergency medicine, this improved performance cannot be attributed to a higher level of previous knowledge in the intervention group. This defuses the argument that those developing OSCE stations in this study were higher performing, more enthusiastic students. Moreover, the argument that familiarity with the OSCE format or practice at a number of stations may have resulted in a generalizable improvement is refuted by the better results of the intervention group than the test OSCE group.

Instead, the intervention group’s better exam results can most likely be explained by the greater amount of time they spent addressing practical content of emergency medicine. This positive learning effect has already been described for students generating MC questions and is therefore referred to in this context as a 'generation effect’ [10,11].

The hypothesis that the intervention group students did better in the exam because they had dealt with practical aspects of emergency medicine more intensively is backed up by the fact that they tended to score higher at all OSCE stations (Figure 1). In fact the members of the intervention group had begun working on practical elements of emergency medicine two months before the start of emergency medicine training according to the syllabus. In our view, the main effect of the teaching intervention is therefore accounted for by the intervention group’s active, more thorough study of practical emergency medicine overall and is not related to a contamination bias due to previously known OSCE stations. Future studies could quantify the learning effort by using learning diaries to better understand the process induced by the intervention [17].

The key finding of our study is its potential impact on training. Students who are interested in assessment-related content (such as emergency medicine in our study) could systematically be involved in the development of OSCE stations early on, as they would benefit both during their initial medical degrees and in their subsequent specialist training. A recent quantitative study proposed a framework for the factors impacting on students’ pre-assessment learning [18]. However, this model does not take the preparation of assessment materials itself into account and it might be supplemented by this task type in 'the creation of assessment items’ in the future.

Another advantage of encouraging students to develop OSCE stations is the potential to cut the faculty’s workload in preparing OSCEs. After all, one reason why OSCEs are currently rarely used in Germany is probably because they tie up considerable time and resources [7]. The logistical and financial outlay deters many faculties from holding OSCEs, especially since the resources required for oral and MC exams are comparatively low [19,20]. But the preparatory work for an OSCE could be minimized if students were involved in its planning, especially the development of scenarios and stations. OSCE stations could be developed not just by students preparing for the regular emergency medicine exam but also by more senior students (e.g. during their internships) in order to refresh their skills and knowledge in emergency medicine.

Although not stated as a research question, another interesting finding of our study is the similarity of the faculty-developed OSCE and student-developed OSCE stations. Six stations were nearly identical (advanced life support, peripheral venous access, bag valve mask ventilation, neck immobilization, chest pain and newborn reanimation). Merely two stations designed by the students were not regarded as essential by the faculty staff (insertion of a central venous line and endotracheal tube insertion). The similarity between OSCE stations reflects the 'constructive alignment’ of the emergency medicine curriculum. Learners construct their own learning through relevant learning activities. The teacher’s job is to create a learning environment that supports the learning activities appropriate to achieving the desired learning outcomes. The key is that all components in the teaching system – the curriculum and its intended outcomes, the teaching methods used and the assessment tasks – are geared to each other [21].

Moreover, in our opinion the quality of the student-developed OSCE stations did not differ substantially from the faculty-developed ones. This statement might, however, be a little speculative since there was no deliberate, standardized analysis of quality or similarity. This point should be addressed by future studies. Nevertheless, these findings further underline the potential of involving interested students in the development of their assessment.

### Limitations of the study

In the final analysis, it remains difficult to distinguish whether generalizable improvement results from more practice on a number of stations or spending more time on practical aspects of emergency medicine by students creating OSCE stations. This is especially true for the comparison between the intervention and control group. However, the fact that the students of the intervention group scored better than the test OSCE group clearly supports the argument that a positive learning effect arose from the voluntary creation of OSCE stations. Alternatively, the study could be repeated with experienced test-takers rather than OSCE novices, because the practice effect on OSCE novices is probably bigger.

Moreover, the study was conducted monocentrically and only for an OSCE in emergency medicine, begging the question of its validity for other medical content domains. However, since the 'generation effect’ has been proven for MC questions [10,11], the generation of OSCE stations can be assumed to bring about a similar effect. Nevertheless, for these reasons – and also because the number of participants in the intervention group was relatively low – follow-up studies need to be carried out to confirm the findings, preferably in other areas of medicine and with more experienced test-takers than in our study, because the practice effect on OSCE novices is probably greater.

## Conclusions

Students who themselves develop OSCE stations in emergency medicine perform better in the subsequent clinical summative OSCE. This finding is a good argument for involving interested students in the development of their assessment.

## Competing interests

The authors declare that they have no competing interests.

## Authors’ contributions

WH participated in the conception and design of the study, carried out the data acquisition. In addition he participated in the analysis and interpretation of data and he drafted the manuscript. DR and GH participated in the data acquisition. MZ participated in the conception and design of the study as well as in the analysis and interpretation of data. PS and UK critically reviewed the manuscript. MF participated in the conception and design of the study as well as in the analysis and interpretation of data. He critically reviewed the manuscript. All authors read and approved the final manuscript.

## Pre-publication history

The pre-publication history for this paper can be accessed here:

http://www.biomedcentral.com/1472-6920/13/138/prepub
